# Supplementary material for: The Systems Biology Research Tool: evolvable open-source software
Source: BMC Syst Biol. 2008 Jun 29;2:55. doi: 10.1186/1752-0509-2-55 (PMC2446383; doi:10.1186/1752-0509-2-55)
Supplement: Additional file 1 — SBRT Archive. An archive of the current version of the Systems Biology Research Tool. [file 1752-0509-2-55-S1.zip › sbrt-1.4.0/doc/developers_guide/api/sbrt/shell/io/FileFormat.html]

FileFormat


|  |  |  |
| --- | --- | --- |
| **Overview** \| **Package** \| **Class** \| **Use** \| **Tree** \| **Deprecated** \| **Index** \| **Help** | |  |
| **PREV CLASS**   **NEXT CLASS** | **FRAMES**    **NO FRAMES**     **All Classes** |  |
| SUMMARY: NESTED \| FIELD \| CONSTR \| METHOD | DETAIL: FIELD \| CONSTR \| METHOD |  |


---


## sbrt.shell.io Class FileFormat

```
java.lang.Object
  sbrt.shell.io.FileFormat
```

---

``` public class FileFormat extends java.lang.Object ```

This class is used to represent file formats.
A file format is a particular way to encode
information for storage in a computer file.

Instances of this class are immutable.

**Author:**
:   This class was written and documented by
    Jeremiah Wright while in the Wagner lab.

---

| **Field Summary** | |
| --- | --- |
| `static FileFormat` | `GZIP_SER`             Used to denote a file containing serialized objects that has been compressed using the GNU program gzip. |
| `static FileFormat` | `GZIP_TEXT`             Used to denote a plain-text file that has been compressed using the GNU program gzip. |
| `static FileFormat` | `SERIALIZED`             Used to denote a file containing serialized objects. |
| `static FileFormat` | `TEXT`             Used to denote a plain-text file. |


| **Method Summary** | |
| --- | --- |
| `java.lang.String` | `getName()`             Returns the name of this file format. |
| `java.lang.String` | `toString()`             Returns a string representation of this file format. |

**Methods inherited from class java.lang.Object**

`clone, equals, finalize, getClass, hashCode, notify, notifyAll, wait, wait, wait`

**Field Detail**

### TEXT

```
public static final FileFormat TEXT
```

:   Used to denote a plain-text file.

---


### GZIP\_TEXT

```
public static final FileFormat GZIP_TEXT
```

:   Used to denote a plain-text file that has been
    compressed using the GNU program gzip.

---


### SERIALIZED

```
public static final FileFormat SERIALIZED
```

:   Used to denote a file containing serialized objects.

---


### GZIP\_SER

```
public static final FileFormat GZIP_SER
```

:   Used to denote a file containing serialized objects
    that has been compressed using the GNU program gzip.


**Method Detail**

### getName

```
public java.lang.String getName()
```

:   Returns the name of this file format.

    :   **Returns:**: the name of this file format.

---


### toString

```
public java.lang.String toString()
```

:   Returns a string representation of this file format.

    :   **Overrides:**: `toString` in class `java.lang.Object`
    :   **Returns:**: a string representation of this file format.


---


|  |  |  |
| --- | --- | --- |
| **Overview** \| **Package** \| **Class** \| **Use** \| **Tree** \| **Deprecated** \| **Index** \| **Help** | |  |
| **PREV CLASS**   **NEXT CLASS** | **FRAMES**    **NO FRAMES**     **All Classes** |  |
| SUMMARY: NESTED \| FIELD \| CONSTR \| METHOD | DETAIL: FIELD \| CONSTR \| METHOD |  |


---
